# Toxic or Otherwise Harmful Algae and the Built Environment

**DOI:** 10.3390/toxins13070465

**Published:** 2021-06-30

**Authors:** Wolfgang Karl Hofbauer

**Affiliations:** Umwelt, Hygiene und Sensorik, Fraunhofer-Institut für Bauphysik, 83626 Valley, Bavaria, Germany; wolfgang.hofbauer@ibp.fraunhofer.de

**Keywords:** phycotoxins, cyanotoxions, cyanobacteria, Chlorophyta, aerophytic algae, anthropogenic structures, chlorellosis, building relevant organisms, microbial induced corrosion, allergenic algae

## Abstract

This article gives a comprehensive overview on potentially harmful algae occurring in the built environment. Man-made structures provide diverse habitats where algae can grow, mainly aerophytic in nature. Literature reveals that algae that is potentially harmful to humans do occur in the anthropogenic environment in the air, on surfaces or in water bodies. Algae may negatively affect humans in different ways: they may be toxic, allergenic and pathogenic to humans or attack human structures. Toxin-producing alga are represented in the built environment mainly by blue green algae (Cyanoprokaryota). In special occasions, other toxic algae may also be involved. Green algae (Chlorophyta) found airborne or growing on manmade surfaces may be allergenic whereas Cyanoprokaryota and other forms may not only be toxic but also allergenic. Pathogenicity is found only in a special group of algae, especially in the genus Prototheca. In addition, rare cases with infections due to algae with green chloroplasts are reported. Algal action may be involved in the biodeterioration of buildings and works of art, which is still discussed controversially. Whereas in many cases the disfigurement of surfaces and even the corrosion of materials is encountered, in other cases a protective effect on the materials is reported. A comprehensive list of 79 taxa of potentially harmful, airborne algae supplemented with their counterparts occurring in the built environment, is given. Due to global climate change, it is not unlikely that the built environment will suffer from more and higher amounts of harmful algal species in the future. Therefore, intensified research in composition, ecophysiology and development of algal growth in the built environment is indicated.

## 1. Introduction

In this review the question of aerophytic algae that is potentially harmful to humans occurring in the built environment shall be pursued. If not stated differently, the term “algae” stands for eukaryotic algae and cyanoprokaryota together. It is common knowledge that algal blooms from marine or fresh water bodies may cause harm to men and livestock due to the production of toxic compounds. The relations may be different in terrestrial ecosystems and the built environment. In the course of the review, it became clear very quickly that the discussion may be divided into several different subtopics, that are treated in the text by the following headings: toxic algae in the narrow sense, allergenic algae, potentially pathogenic algae and algae toxic to buildings and works of art.

### 1.1. Where Do Aerophytic Algae, Potentially Harmful, Exist in the Human Built Environment?

Aerophytic algae are very widespread and are colonizing diverse habitats on land. They are also found on building surfaces exposed to climate action and may be recognized as a nuisance. Depending on the local micro- and nanoclimates, different manmade materials may be colonized in different amounts and many species have been isolated and/or identified from anthropogenic substrates so far. In recent studies on the initial growth on modern façades, an unexpected diversity was found with more than 70 species. On surfaces of insulated façades, eukaryotic algae, especially green algae prevail, mainly Trebouxiophyceae, supplemented by other algal taxa [1,2,3]. Substrates and habitats may be all exposed materials, masonry, concrete, metal posts and signs, varnished surfaces, plastic, glass, ceramics, wood, monuments and other works of art, etc. Algae growing on buildings and trees may also contribute significantly to the total atmospheric algal load [4]. However, not only material exposed to outdoor conditions may be colonized by algae, but also sheltered habitats indoors, if conditions are favorable. Additionally, we also have to consider the not so obvious constituents of the built environments such as drainage and sewage systems and of service facilities. The well-known so called “Lampenflora” in caves and tunnels flourish on damp surfaces with artificial light sources only [5,6]. In Table 1, an overview on potentially harmful airborne algae compared to algae occurring in the built environment is given, extracted from a choice of different work (paragraph 6).

### 1.2. How Does Man Get in Contact with Potentially Harmful Aerophytic Algae?

Along with other microorganisms, algae are found in the atmosphere. In pioneering investigations in 1844, Ehrenberg identified as a first algae from aerial dust probes [7] collected previously by Darwin. The aerial environment approximately carries half of the global microbial diversity according to [8]. Aerophytic algae, algae that grows on substrates exposed to the atmosphere, often are dispersed via air currents and thus become airborne. This means of transport via the air is very important for aerophytic algae as is the ground-near aerosol that is produced during precipitation by splashing raindrops [2,3,9,10]. In rain, incidences also from the surface of freshwater bodies’ algae may be aerosolized [10,11,12]; the same also happens through the bursting of bubbles and the breaking of waves, comparable to processes in marine waves [10,13,14]. In [15] it was determined that, therefore, an amount of up to 1.6 × 10^5^ cells per m^3^ of picocyanobacteria may be aerosolized from freshwater. Gregory et al. [16] measured high numbers of cells of a *Gloeocapsa* sp. in the air from several locations in Great Britain (an average of 110 *Gloeocapsa* cells/m^3^). Since 1844, according to [10,17], a total of more than 350 taxa (genera and species) have been identified in aerobiological studies [7,16,18,19,20,21]. Such aerosols can easily reach the human organism and may provoke a reaction, e.g., if inhaled or deposited on the skin [8,17,22,23,24]. It has been estimated that humans may inhale approximately 1500 algal cells per day [25,26], or even more [18]. However, it is still unclear how big the amount of microorganisms (species dependent) that must be inhaled is to cause adverse health effects. Inhalation of airborne cyanobacteria and microalgae can lead to allergies, rhinitis, asthma, bronchitis, and dermatitis or intoxication [17,22]. Another form of direct contact can be through the skin if it rubs over a surface colonized by algae. Accidental ingestion may be possible (e.g., drinking water). In addition to the ways aerophytic algae may get into contact with the human organism, they may be spread within the built environment by different human activities. Anthropogenic spread has been shown impressively for remote and/or new ecosystems such as the Antarctic [27].

### 1.3. How Can Potentially Harmful Aerophytic Algae Be Detected and Assessed?

Airborne and aerophytic algae occurring in the built environment may be detected in various ways, depending on the substrate (air, surface, water body or building material). Regarding the sampling and monitoring of aerosolized algae, quite a range of methods exist: e.g., fan dust sampling, filtration, impaction, impingement, Rotorod sampling, sedimentation, application of a vacuum cleaner and wind nets, etc. In addition, for collecting samples from surfaces, diverse qualitative and quantitative methods have been achieved, as discussed in [3,28]. If not only quality but also quantity shall be assessed, the source must be quantifiable, as is possible, e.g., with a filtration method for air sampling supplemented by dilution plating. Whatever methods are applied, the accurate identification of the observed forms is always crucial, especially when focussing on potential harmful species. In numerous works on algal growth on manmade surfaces, many of different species have been found. Many of them comprise difficult taxa that are not easy to distinguish—therefore, the value of cultures as a major tool for taxonomic analyses cannot be overstressed [3,29,30]. Recently, a culture collection focusing on the built environment, BRMO—building relevant micro-organisms—has been established at the Fraunhofer Institute for Building Physics (IBP), mainly based on proprietary investigations, with emphasis on aerophytic algae and fungi [1,2,3,31]. Increasingly, molecular methods are adopted and applied as essential tools in assessing the microbial communities of the built and urban environments [32,33,34], lately also metagenomics approaches [35,36]. Still, cultures are indispensable in the elucidation of effects and the potential of aerophytic algae occurring in the built environment as well as their taxonomic affiliations and their relation to other organisms. In addition, advanced microscopic and spectrometric techniques can be employed. Applying modern digital microscopic and photographic techniques, image analysis of building surfaces facilitates the assessesment of growth patterns and intensity [37,38,39,40]. To quantify algal growth on concrete and similar surfaces, a special method for chlorophyll extract measurement has been adopted [39]. Chlorophyll quantitation was already used otherwise to assess algal density on manmade structures, as well as chlorophyll fluorescence [41,42,43]. Confocal microscopy may reveal the colonization process and the three-dimensional structure of the crust community [44,45,46] in very great detail, although it is not yet possible to assess the taxonomic affiliation of the observed organisms accurately.

## 2. Toxic Algae Occurring in the Built Environment

There is a variety of algal organisms that are capable producting a wide array of toxic substances (phycotoxins) and allelochemicals. If such algae reach a state of a mass development, their toxin production becomes evident and may have severe consequences. Generally, it is assumed that phycotoxins are involved in some defense processes and therefore producers put up with additional metabolic costs. Recently, this assumption was challenged by the work of [47]. Their results show that under normal environmental conditions, phycotoxin production in Dinophyceae might cause no significant intrinsic growth rate costs, and they suggest future research in understanding the evolutionary role and ecological function of algal toxins.

Poisonous algae blooms in marine environments (“red tides”) typically produced from dinoflagellates (Dinophyta) are well known and dreaded. Especially red tide toxins (e.g., brevetoxins, ichthyotoxin and related compounds) are notorious for their neurotoxicity and the potential accumulation in shellfish (“neurologic shellfish poisoning”). Toxins produced from aerophytic or otherwise extremophile algae are discussed under various aspects in great detail in [48]. Therefore, in the following paragraphs, in toxigenic algae and their toxins a potential connection to the anthropogenic environment shall be stressed.

Whereas red tides and shellfish poisoning are incidences directly connected to the marine environment, it was observed that aerosolized marine algal toxins may also be harmful to men. One of the first incidences of aerosolized algal toxins might have been reported by [49], who described human respiratory irritation associated with high concentration of plankton (“red water”) in Florida and mass mortality of marine organisms. The marine dinoflagellate *Karenia brevis* (*Gymnodinium brevis*) is responsible for such red tides that form in the Gulf of Mexico, producing brevetoxins. Brevetoxins are transferred from marine water to air through white-capped waves during red tide episodes [50]. When aerosolized, the toxins cause airway symptoms (e.g., asthma) in normal individuals and patients with airway disease and also lead to (allergic) skin reactions [50,51,52,53]. Inhalation of brevetoxins could be shown in animal models and probationer/patient trials [51,54,55].

Palytoxin, another toxic substance originating from algae, is considered to be one of the most poisonous non-protein substances known. It is produced from the dinoflagellate *Ostreopsis siamensis* and Cyanoprokaryota of the genus *Trichodesmium*. The compound can be concentrated in marine animals such as fish or Anthozoa, e.g., *Palythoa toxica*, thus the name “Palytoxin”. Exposures have happened in people who have eaten sea animals such as fish and crabs or who got into contact via the skin, e.g., who have handled *Palythoa* corals incorrectly [56,57]. Cases of inhalation are known, as demonstrated in 2005 by a mass poisoning of people by marine aerosol [58]. There are several reports on hospitalized people by inhalation of palytoxins released during removal or handling of corals from personal aquariums—these cases clearly representing incidents happening in the built environment, even indoors [59,60,61,62].

Other examples of algal toxins (phycotoxins), typically produced by marine dinoflagellates, are maitotoxin, an extremely potent toxin produced by *Gambierdiscus toxicus*, a dinoflagellate species which also produces ciguatoxines; and dinophysistoxins with okadacid-derivates mainly produced by *Dinophysis* spp. Further examples of toxic species are known from the various Dinophyta genera, e.g., *Amphidinium*, *Cochlodinium*, *Gymnodinium*, *Gyrodinium* and *Prorocentrum*. *Gymnodinium*, *Gyrodinium* and *Prorocentrum* are also known as potentially harmful airborne algal taxa, and *G. tenuissimuim* was recorded for Lampenflora [5,17] (Table 1). With global climate change, it is assumed that severity and frequency of red tides will increase [63]. Whether this will lead to more abundant occurrences of Dinophyceae in the built environment remains unclear for now.

The discussed dinoflagellates are the principle producers of phycotoxins; however, apart from that there are known toxigenic diatoms, euglenophytes, raphidophytes, green algae, cyanobacteria and prymnesiophytes, etc. *Prymnesium parvum* may form fresh water or estuary blooms that are devastating to fish. *Prymnesium* blooms recently have occurred in previously unaffected regions, such as Hungary and North America, but there are no records on humans being affected so far [64,65,66,67].

Some diatoms (Bacillariophyceae) from the genus *Pseudonitzschia* may form toxic blooms in the sea with the active compound domoic-acid leading to amnesic shellfish poisoning. Exposure to domoic-acid affects the brain via damages especially in the hippocampus and amygdaloid nucleus, causing short-term memory loss, seizures, kidney failure and possibly death [68,69]. Diatoms are known from the built environment [3,70]. *Amphora* spp. (domoic acid) and *Licmophora* spp. (unknown allelochemicals) are registered as potential harmful airborne algal taxa [17]. However it seems not very likely that heavy toxic diatom blooms may occur at buildings or the built environment so far. On the other hand, it is long established knowledge that diatoms may be aerosolized [7].

Another group of eukaryotic algae with secondary plastids such asDinophyceae and Bacillariophyceae that may produce toxins are Euglenophyceae. Euglenophycin, a toxin produced in freshwater by *Euglena sanguinea* was first recognized after a fish mortality event in North Carolina [71,72,73]. Recently, even more species of Euglenophyceae were reported to be able to produce Euglenophycin. Species producing Euglenophycin in significant amounts include *Lepocinclis acus*, *Trachelomonas ellipsoidalis*, *Strombomonas borysteniensis*, two species of *Euglenaria* (*E. clavata, anabaena*) and three species of *Euglena*—*E. sanguinea, sociabilis, stellata* [74]. Exposure to E. may cause fish deaths within only two hours [71]. Although harmful in fish, the toxin is also discussed as a potential compound useful in cancer therapy [75]. Although some Euglenophyceae may live aerophytically, there are no toxic forms known to occur aerophytically in the built environment so far [30]. However, indoors, a *Euglena* sp. has already been found (Table 1); since the species could not be revealed, a toxic form cannot be excluded with certainty.

Raphidophyceae are forming a special group of heterokont algae, mostly flagellates typically thriving in acid fresh or marine water. *Fibrocapsa japonica* is a species that was discovered only in 1973 at the coasts of Japan, which may produce neurotoxins [76]. These toxins may have a lethal effect on fish [77]. Rapid growth (blooms) of this species may become a serious threat to the Japanese inshore fishing industry. The same species has been occurring from the 1990s onwards, with growing abundance at European shores. In 1995, it was also detected in the German Wattenmeer [78]. Some species of the marine genus *Chattonella* may also produce toxins harmful to fish [79]. Other species such as *Gonyostomum semen* are known freshwater toxin producers (neurotoxins) in water blooms. The increasing acidification of waters attributed to climate change may lead to a spread of such forms, with effects on small water bodies as well [80]; thus, they might even reach cities.

Although widespread in limnic as well as soil and other aerophytic habitats, there is no documented case of a toxic member of the Xanthophyceae [81].

Much more abundant in the built environment than the previous discussed groups are green algae and Cyanoprokaryota. Toxic species are also known in green algae (Chlorophyceae). The most notorious is the marine invasive killer alga, *Caulerpa taxifolia*. It was found that the alga contains a toxin in its thallus lobes, Caulerpenyne, which is noxious (neurotoxic) to various animals, especially invertebrates [82,83]. Originally dreaded as a potential danger to natural biodiversity, it was found the opposite; the alga reduced pollution and aided in the recovery of native *Posidonia* seagrass [84]. In [81], for several Caulerpa species, two other toxins are noted, namely caulerpicin and caulerpin.

Ref. [85] reported that the planktonic freshwater alga *Botryococcus braunii* (Chlorophyta, Trebouxiophyceae) has toxic effects on aquatic organisms. Blooms of this alga are associated with fish deaths. Experiments revealed that free fatty acids produced by the alga, particularly oleic and α-linolenic acids, are functioning as allelochemicals. Fatty acids seem also to be involved in ichthyotoxic activity of the freshwater chlorophycean alga *Chaetomorpha minima* (Chlorophyta, Ulvophyceae) [81]. Fatty acids are also responsible for antibacterial and allelopathic features of *Haematococcus pluvialis* (Chlorophyta, Chlorophyceae; also known from the built environment) and *Skeletonema costatum* (Bacillariophyta, Mediophyceae), otherwise evident as toxic algae species, according to [86]. Refs. [86,87,88] give further information on the antimicrobial activities of microalgae. Allelochemicals and/or antibacterial compounds are known from some taxa of green algae that occur aerophytic or on surface crusts in the built environment: e.g., in *Chlorella vulgaris* (Chlorellin and unidentified bioactive compounds)*, Auxenochlorella* (*Chlorella*) *pyrenoidosa* (Chlorellin), *Chlorococcum infusionum* (fatty acids and unidentified bioactive compounds), *Desmococcus olicaceus* (unidentified bioactive compounds), *Stichococcus bacillaris* (probably unsaturated fatty acids and unidentified bioactive compounds) and except *Chlorococcum* (Chlorophyta, Chlorophyceae), all members of the Trebouxiophyceae [89,90,91,92,93,94,95,96,97].

So far there is scarce evidence that toxic green algal blooms flourish extensively on building surfaces outdoors or indoors. However, the mentioned examples clearly show that members of the Trebouxiophyceae, which form an important part in the colonization of building materials [3], are capable of the production of bioactive substances potentially toxic to other organisms.

Cyanoprokaryota (blue-green algae) are distributed in water (fresh, brackish, and marine), terrestrial and aerophytic environments throughout the world. Under favourable conditions, excessive growth such as bloom formation, especially in fresh water basins or coastal waters, of certain Cyanoprokaryota develops. Cyanoprokaryota produce a great spectrum of secondary metabolites which can be toxic in relevant amounts in animals and humans (cyanotoxins). *Nodularia* sp. perhaps was the first toxic cyanoprokaryota alga reported in literature [98,99]. For various species and groups of species, different toxins and derivate compounds are known. Humans can be exposed in different ways, mainly orally via drinking water or consumption of contaminated food such as algal health food tablets. Accumulation in aquatic organisms and crop plants has been demonstrated [100]. Further exposure routes are dermal contact and accidental inhalation of aerosol or accidental ingestion via, e.g., the recreational use of contaminated water bodies [101]. The effects of cyanotoxins have been known for more than 120 years now, when the death of cattle was attributed to their drinking of water during algal bloom [102]. However, the cutaneous and adverse effects of Cyanoprokaryota and their cyanotoxins are still often underdiagnosed [103]. Consequently, there is a long-standing lack of knowledge regarding the adverse cutaneous and inhalative effects of Cyanoprokaryota and their toxins, although seaweed dermatitis (contact dermatitis) was the first described cutaneous reaction, which was recognised after contact with toxic Cyanoprokaryota bloom in marine waters [104,105].

Microcystins (MC’s, of “fast death factor” FDF [81]) are cyclic heptapeptides estimated to vary between 500 and 4000 Da, and 64 variants have been described so far [106,107]. Microcystins (MC’s) form the main family of cyanotoxins since they are the most frequent and most widespread. MC’s were first isolated from *Microcystis aeruginosa* and are found in most populations of *Microcystis* spp., *Anabaena* spp. (*Dolichospermum*), *Anabaenopsis* spp., *Aphanocapsa* (*cumulus*), *Nostoc* spp., *Oscillatoria* (*Planktothrix*) (*P. agardhii*, *P. rubescens* and *O. tenuis*) and a soil isolate of *Hapalosiphon hibernicus* [107]. Once absorbed, MCs concentrate in the liver where they exercise hepatotoxic effects. Most of the human poisonings were limited to gastro-enteritis, but fatal cases also happened [108,109,110]. Illness in humans associated with inhaling microcystins has been documented [107,111]. In addition, poisonings including allergic reactions have been recorded [112]. Recently, harmful algal blooms dominated mainly by *Microcystis* spp. occurring in urban ponds were reported [113].

Aplysiatoxins are phenolic bislactones and are known, e.g., from *Lyngbya majuscula*, *Schizothrix calcicola* and *Oscillatoria nigro-viridis* [107,114]. Aplysiatoxins are strong skin irritants, if ingested they were also involved with poisonings causing diarrhoea and burning sensations of the mouth and throat [107,115].

ß-N-methylamino-L-alanine (BMAA) was identified only recently, but already found in diverse Cyanoprokaryota [116]. It comprises a non-protein amino acid that acts mostly on motor neurons by fixation on glutamate receptors [101]. Further toxicological data are lacking, but there are assumptions that BMAA could be associated with various neurodegenerative diseases [101,117,118,119].

Saxitoxins (STX’s) are a group of carbamate alkaloid toxins possessing a unique tricyclic structure with hydropurine rings occurring in various cyanobacteria [107,120]. Saxitoxins may be found in *Anabaena* spp., mainly in *Dolichospermum circinale* (*A. circinalis*), but also others, e.g., *Dolichospermum perturbatum* (*A. spiroides* var. *tumida*), *Dolichospermum spiroides* (*A. spiroides*), *Dolichospermum lemmermannii (A. lemmermannii)*, *Dolichospermum flos-aquae* (*A. flos-aquae*), etc.; *Plectonema wollei* (*Lyngbya wollei*); *Oscillatoria* (*Planktothrix*) spp.; and *Cylindrospermopsis raciborskii* [107,121,122,123,124]. In seawater, Saxitoxins are also produced by some dinoflagellates [101]. Saxitoxins, also known as paralytic shellfish poisons, have been associated with numerous human intoxications resulting in numbness, complete paralysis and even death [110]. To date, no reports on poisonings in freshwater environments due to Saxitoxins in humans are known [107].

Anatoxin-a’s (“very fast death factor VFDF” [81]) with derivates/homologues are low molecular weight tropane related alkaloids and structural analogues of cocaine [107]. They are known from *Anabaena* spp. (inclusive *Dolichospermum circinalis*), *Oscillatoria* spp. (inclusive *Planktothrix* spp.), *Cylindrospermum* spp., *Aphanizomenon* spp., *Tychonema* spp. and *Raphidiopsis mediterranea* and may occur in minor amounts in *Microcystis* spp. [106,107,125]. Anatoxin-a’s induce paralysis, and consequently, death can occur by respiratory arrest [101,125]. So far there is no information available on toxicity to humans [101,107]. Anatoxin-a may also be produced by *Phormidium autumnale*, a taxon that is widespread, also in the built environment [126].

Anatoxin-a(S), unrelated to anatoxin-a, comprises a unique guanidinium methyl phosphate ester, becomes inactivated at elevated temperatures and was found only in planktonic *Anabaena* species so far. Anatoxin-a(S) induces muscular paralysis with potential death by respiratory arrest [101], but no reports on poisoning of humans are available.

Further cyanotoxins are hepatotoxic nodularins, so far only known from *Nodularia* spp. and *Iningainema* spp. [127]. Hepatotoxic cylindrospermopsins have so far only been isolated from *Cylindrospermopsis raciborskii*, *Umezakia natans*, *Chrysosporum ovalisporum* (*Aphanizomenon ovalisporum*), *Chrysosporum bergii* (*Anabaena bergii*) and *Rhadiopsis curvata*. Moreover, lyngbyatoxins are so far only known from *Lyngbya majuscula*, which is a marine algal form. All of the above toxins are not known to have adverse effects on man in connection to limnic or terrestric habitats or have not been found to cause problems in an aerosolised form [107].

Tubercidin and other 5′-α-D-glucopyranose derivatives of the nucleosides are the major cytotoxins of some aerophytic filamentous Cyanoprokaryota belonging to the Scytonemataceae, including *Hassallia byssoidea* (*Tolypothrix byssoidea*) [128], which was already recorded for building surfaces.

Lipopolysaccharides (LPS) from Cyanoprokaryota are discussed as potentially (irritant) poisonous compounds [129,130]. In Table 1, important toxins produced in the listed taxa with emphasis on airborne or aerophytic forms are given, as available from literature, also partly considering further toxic compounds or compounds under suspicion to be toxic not discussed above.

Due to their inherent characteristics, Cyanoprokaryota typically need liquid water to be physiologically active and their aerophytic forms can usually cope with prolonged desiccation. Cyanoprokaryota prefer tropical/subtropical or arid geographical regions or habitats with permanent or recurring precipitation. Reviewing cyanotoxins, it appears that microcystins, aplysiatoxins and BMAA’s could be the most likely cyanotoxins that could appear in the built environment even at surfaces exposed to air or in small water bodies due to the presence of organisms that are potential toxin producers in the built environment. So far no incidents have been documented of poisonings triggered by Cyanoprokaryota that can be connected directly to the built environment. Still, new cyanotoxins are being discovered, as mentioned in [131], for the marine environment. Further scientific work is thus necessary. Finally, it has to be recognised that Cyanoprokaryota are producing not only strictly harmful secondary metabolites, but some of their defence compounds may also be used beneficially in the fight against other harmful organisms [132].

Usually, eukaryotic algae and cyanoprokaryota forming biogenic crusts on building materials are growing in mixed communities where algae are not the only components but are accompanied by other organisms as well, such as (other) bacteria, fungi, lichens, mosses and sometimes also higher plants and even animals [3]. Especially accompanying fungi might be interesting in the discussed question because many of the so-called mould fungi are known producers of toxins, i.e., mycotoxins. Examples for toxigenic fungi reported from the built environment are members from the genera *Stachybotrys* (toxic mould), which may produce satratoxins and other trichothecenes [133], and *Fusarium*, whose numerous species may secrete various toxic substances such as zearalenone, fumonisins, moniliformin, trichothecenes, etc. [134]. In addition, mycotoxins may get aerosolized or transported via spores and hyphal fragments through the air, causing health problems in humans; upon contact with sensitive surfaces of the body, e.g., the eyes and the interior of mouth or nose, inflammation may occur [135,136].

Studying the diversity of algae occurring on buildings, species have been identified that are known for their toxin producing ability (Table 1). Even if such species do occur, the overall biomass normally produced by algae on building surfaces in temperate climates is comparable low. In contrast, in (sub)tropical areas, algal growth on building surfaces might be much more luxuriant. In these areas, and in (small) bodies of water present in the built environment, blooms of potentially toxic algae might occur and express a potential health hazard if ingested, touched or if aerosolized and inhaled. Freshwater algal blooms are occurring worldwide with increasing incidence [21,111], which means that the connected dynamics will intensify in the future. Furthermore, many species of algae that may occur in the built environment are still not documented sufficiently and many compounds that are produced by aerophytic algae are not elucidated so far. What we certainly know is that the relations in biogenic crusts and biofilms are very complex and involve numerous chemicals, especially compounds with allopathic or otherwise regulative characteristics. Therefore, it is quite certain that many more bioactive compounds will be found in the future in algal crusts on buildings. With further global climate change, there will also be a shift in biodiversity regarding the built environment. Thermotolerant species may prevail in regions with elevated ambient temperatures. It is well known that the physiological activity of organisms increases with temperature (until it reaches lethal dimensions), also involving increased production of secondary compounds such as toxins. This is already documented for toxic Cyanoprokaryotes and their cyanotoxin production [137]. Marine and fresh water algal blooms are triggered by global warming and eutrophication. Frequency, intensity as well as a shift poleward of algal blooms may result. The first signs of such processes have been observed already. In artic marine environments, potential toxic blooms have emerged, and toxic cyanoprokaryota are proliferating in freshwater environments [63,138,139]. Considering these aspects, it is not unlikely that in the future the built environment might suffer from more toxic species and higher amounts of toxins produced. Therefore continuous observation of the composition and development of biogenic crusts of the built environment seems feasible.

**Table 1:** Potentially harmful airborne algae, potential health implications and important toxins set into context with algal forms occurring indoors, in Lampenflora and on building surfaces. BMAA: β-N-methylamino-L-alanine; MIB: 2-methylisoborneol; LPS: Lipopolysaccharides; p.p.: per parte; s.l.: sensu lato; spp.: species plural; +: taxon recorded in any of the searched publications; (?): record not conclusive.

Potential Harmful Airborne Algal Taxon [8,10,17,19,23,26,140,141,142]	Potential Health Implications and Major Toxins of Toxigenic Species [17,26,74,107,116,129,130,143,144,145,146,147,148,149,150,151,152]	Potential Harmful Airborne Algae Found Indoors [142,153,154,155,156,157,158]	Potential Harmful Airborne Algae Occurring in Lampenflora [5,159,160,161,162]	Potential Harmful Airborne Algae Growing on Building Surfaces [3,163,164,165,166,167,168,169,170,171]
Chlorophyceae				
*Ankistrodesmus* spp.	Allergy			
*Ankistrodesmus falcatus*	Allergy	+	+	
*Bracteacoccus* spp.	Allergy			+
*Chlamydomonas* spp.	Allergy, Dermatitis, Rhinitis, Asthma	+	+	+
*C. agloëformis*	Allergy?			
*Chlorella* spp. s.l. (inclusive e.g., *Auxenochlorella*, *Chloroidium* p.p., *Mychonastes* p.p.)	Allergy, Rhinitis, Hyper-sensitivity	+	+	+
*Chloroidium saccharophilum* (*Chlorella saccharophila*)	Allergy(?)			+
*Auxenochlorella* (*Chlorella*) *pyrenoidosa*	Allergy			
*Chlorella vulgaris*	Allergy	+	+	+
*Chlorococcum* spp.	Allergy	+	+	+
*Chlorococcum diplobionticum*	Allergy(?)			
*Chlorococcum ellipsoideum*	Allergy(?)			+
*Chlorococcum infusionum*	Allergy	+	+	+
*Chlorosarcinopsis* spp.	Allergy			+
*Coccomyxa* spp.	Allergy		+	+
*Coccomyxa confluens*	Allergy, Dermatitis, Rhinitis, Asthma			
*Myrmecia* spp. s.l.	Allergy, Dermatitis, Rhinitis, Asthma			+
*Neochloris* spp. s.l. (inclusive *Ettlia* p.p., P*arietochloris* p.p.)	Allergy	+		+
*Oocystis* spp.	Allergy	+	+	+
*Palmella* spp.	Fever (Allergy)	+		
*Scenedesmus* spp. s-l. (inclusive e.g., *Desmodesmus* p.p., *Graesiella* p.p., *Tetradesmus* p.p.)	Allergy, Dermatitis,	+	+	+
*Tetradesmus* (*Scenedesmus*) *acutus*	Allergy(?)			+
*Stichococcus* spp. s.l. (inclusive *Pseudostichococcus* p.p.)	Allergy, Dermatitis, Rhinitis, Asthma;	+	+	+
*Stichococcus bacillaris*	Allergenic potential	+	+	+
*Tetracystis* spp.	Allergy			+
*Chlorococcum aerium* (*Tetracystis aeria*)	Allergenic potential			
*Trebouxia* spp. s.l. (inclusive A*sterochloris*, p.p. *Pseudotrebouxia* p.p.)	Allergy, Dermatitis, Rhinitis, Asthma			+
Streptophyceae				
*Klebsormidium* spp.	Allergy	+	+	+
*Klebsormidium subtile*	Allergy(?)			
*Mesotaenium* spp.	Allergy			
*Mesotaenium micrococcum*	Allergy, Dermatitis, Rhinitis, Asthma			
Euglenophyceae				
*Euglena* spp.	Toxin producers, Euglenophycin	+		
Xanthophyceae				
*Xanthonema montanum*	Allergenic potential			+
Bacillariophyceae				
*Amphora* spp.	Toxin producers; domoic acid		+	
*Licmophora* spp.	unidentified allelochemicals			
Dinophyceae				
*Gymnodinium* spp.	Toxin producer		+	
*Gyrodinium* spp.	Toxin producer			
*Prorocentrum* spp.	Toxin producer			
Cyanoprokaryota				
*Anabaena* spp. (inclusive e.g., *Dolichospermum* p.p, *Trichormus* p.p.)	Toxin producers, Microcystins BMAA, Saxitoxins, Anatoxin-a, Anatoxin-a(S), LPS; Allergy, Dermatitis, Rhinitis;	+	+	
*Dolichospermum helicoideum* (*Anabaena helicoidea*)	Toxin producer; Microcystins, Saxitoxins, Anatoxin-a			
*Dolichospermum circinale* (*Anabaena circinalis*)	Toxin producer; Microcystins, Saxitoxins, Anatoxin-a			
*Trichormus fertilissimus* (*Anabaena fertilissima*)	Allergy			
*Anabaenopsis* spp.	Toxin producers, Microcystins			
*Anabaenopsis circularis*	Allergy			
*Arthrospira* spp.	Toxin producer			
*Chroococcus* spp.	Toxin producer LPS	+	+	+
*Cylindrospermum* spp.	Toxin producer; Anatoxin-a	+		
*Gloeocapsa* spp. s.l. (inclusive *Chondrocystis* p.p.).	Toxin producer Microcystins, LPS	+	+	+
*Hapalosiphon* spp.	Toxin producer; Microcystins. LPS	+		+
*Leptolyngbya* spp.	Toxin producers, Microcystins, Coibamide A, Crossbyanols A−D, LPS	+	+	+
*Leptolyngbya fragilis*	Allergy			
*Lyngbya* spp. s.l. (inclusive *Planctolyngbya* p.p.)	Toxin producers, Aplysiatoxins, Saxitoxins, Lyngbyatoxin-a, LPS, Allergy, Dermatitis, Swelling of mucous membranes	+	+	+
*Lyngbya maior*	Allergy			+
*Microcoleus* spp. s.l. (inclusive *Trichocoleus* p.p.).	Dermatitis (Allergy)	+	+	+
*Microcystis* spp. s.l. (inklusive *Aphanocapsa* p.p.)	Toxin producers; Microcystins, BMAA, Anatoxin a; LPS	+	+	+
*Microcystis aeruginosa*	Toxin producer, Microcystins, LPS; Pneumonia			
*Microcystis flos-aquae*	Toxin producer; Microcystins			
*Myxosarcina* spp. s.l. (inclusive *Cyanosarcina* p.p.)	Toxin producers BMAA; Allergy			+
*Nostoc* spp. s.l. (inclusive *Desmonostoc* p.p.)	Toxin producers, Microcystins, BMAA, LPS, unknown Indolocarbazol-compound; Allergy		+	+
*Nostoc commune*	Allergy		+	+
*Nostoc linckia*	Toxin producer, Nostocyclophan D; Allergy			+
Des*monostoc* (*Nostoc*) *muscorum*	Allergy	+	+	+
*Nostoc paludosum*	Toxin producer		+	
*Oscillatoria* spp. (inclusive e.g., *Planktothrix* spp. p.p.)	Toxin producers, Microcystins; Aplysiatoxins, Saxitoxin, anatoxin-a, LPS; Allergy, hay fever	+	+	+
*Oscillatoria simplicissima*	Allergy			
*Phormidium* spp.	Toxin producers, anatoxin a; BMAA; Allergy	+	+	+
*Phormidium angustissimum*	Allergy	+		
*Schizothrix* spp. s.l. (inclusive *Symplocastrum* p.p.).	Toxin producers, Aplysiatoxins, LPS AAA	+	+	+
*Schizothrix calcicola*	Toxin producer, Aplysiatoxins	+	+	+
*Scytonema* spp.	Toxin producers, Saxitoxins, Tolytoxin, Scytophycins, Scytovirin, Scytoscalarol, Scytonemides A and B, LPS	+	+	+
*Scytonema bohneri*	Allergy			+
*Snowella* spp.	Toxin producers, Microcystins, LPS			
*Synechococcus* spp. s.l. (inclusive *Cyanothece* p.p.)	Toxin producers, Microcystins, BMAA, Fatty acids, Linolenic acid, Hemolysins, Lipopeptide, LPS, MIB, Synechobactins A–C, tTionsulfolipid	+	+	+
*Synechocystis* spp.	Toxin producers; Microcystins, BMAA, Anatoxin-a, Fatty acid, LPS, Triterpenoid			+
*Tolypothrix* spp. s.l. (inclusive *Hassallia* p.p.)	Toxin producers		+	+
*Hassallia* (*Tolypothrix*) *byssoidea*	Toxin producer, Tubercidin and other 5′-α-D-glucopyranose derivatives of the nucleosides			+
*Westiellopsis* spp.	Toxin producers			+
*Westiellopsis prolifica*	Allergy Toxin producer, Westiellamide			+
*Woronichinia* spp.	Toxin producers, Anatoxin-a, LPS			

## 3. Allergenic Algae and the Built Environment

Allergies to airborne pollen and fungal spores are a well-known medical issue [172]. An allergic sensitization may develop against various organic and even inorganic substances; therefore it is not extraordinary that algae may also provoke allergic reactions. There are three main ways that allergenic algae may get into close contact with people. First, and certainly most important, people may come in contact with aerosols containing airborne algae or particles and chemical compounds derived from algae; second, via direct contact to the human skin; and third, via ingestion. If we look at the aerial habitat, it becomes obvious that many different species may occur. Schlichting [4,173] reported about 54 taxa of algae in the air; extended by the results of [18] it was 62 genera. Altogether, the range of algal species distributed through the air is certainly much bigger. A total of more than 350 taxa are documented in [10,17].

A first indication on allergenic algae is given by [174], who designated *Palmella*-like forms of soil algae as causes of inter- and remittent fever in Ohio and Mississippi. Unfortunately, the accurate species of the alga cannot be traced, but subsequent investigations by others proved an allergenic potential of algae.

Skin testing for allergic reactions to algae and air sampling for airborne algal cells have resulted in an association of both green algae (e.g., *Chlorella* spp. and *Chlorococcum* spp.) and Cyanoprokaryota (*Schizothrix* spp. and *Anabena* spp.) with adverse human health effects [22,153]. Experimental sensitization of rabbits with green algae *Auxenochlorella pyrenoidosa*, *Chlorella vulgaris* and *Scenedesmus basiliensis* showed different strength of cross reactivity among one another as well as to further green algae: *Chlorococcum botryoides*, *C. macrostigmatum* and *Ankistrodesmus falcatus* [143]. In [140], molecular evidence is given that terrestrial alga do possess allergenic potential, and the focus was especially on *Stichococccus bacillaris* (Chlorophyta), *Tetracystis aeria* (Chlorophyta) and *Xanthonema montanum* (Xanthophyta). In [175], a case of sensitization against a powder of *Chlorella* sp. is reported occurring in a production facility of *Chlorella* tablets in a pharmaceutical factory. Aerophytic green algae (Chlorophyta) may grow under comparable conditions to moulds and are even found as indoor allergens. In an investigation of algae occurring in house dust of 84 patients tested, 58 percent showed positive responses to one or more algal allergens [154]. Ref. [176] refers to a study by her group where, by RAST-testing sera from 33 children, it was shown that 21% were positive for sensitization against *Chlorella* or *Anabaena*. Of the positive children, 57% had one or more aquaria at their home. In another study, 50% mouldallergic children were positive in RAST, containing *Chlorella* specific IgE [177]. In addition to potentially occurring algae house dust is also a source of fungi and mites that may cause severe allergic reactions more commonly reported [178,179]. Although the extent of allergic reactions due to algal exposure has not been fully investigated, house dust and aeration of aquariums have been proposed as possible sources. The clinical relevance of allergenic green algae and yellow-green algae, however, has not been clearly demonstrated yet. Further evidence based research is needed.

On the other hand it was found recently, that *Coenobotrys (Coccomyxa) gloeobotrydiformis* may produce an anti-inflammatory compound that even might act against allergic reactions [180], whereas a different species of the genus *Coccomyxa* is regarded as potentially harmful [17]. In addition, Fucoxanthin, a major accessory pigment in Xanthophyceae and marine Phaeophyceae exhibits qualities beneficial to human health as there are indications that it may positively influence atopic dermatitis [181].

In addition to green and yellow-green algae, further eukaryotic algal taxa may also be involved in allergies. *Gonyostomum semen*, as the most common freshwater raphidophycean, has been reported from Africa, Asia, Europe and North and South America [182,183]. This species often forms blooms in mildly acidic waters and secrets a mucilage that may cause skin irritation and allergic reactions. This phenomenon has led to temporary closure of some freshwater recreational sites [184,185,186]. In recent decades, *G. semen* has rapidly increased its distribution and abundance in lakes in Northern Europe [183,187,188,189], possibly due to processes connected to changed environmental temperatures [190].

Heise [191] reported several cases of sensitization to cyanobacterial blooms in lakes, without a clear outline of the species involved, but the microscopic pictures given makes it clear that filamentous Cyanoprokaryota with sheath had been involved. Apart from the irritant cutaneous effects of cyanotoxins discussed above, hypersensitive immune responses to Cyanoprokaryota were reported as well, primarily regarding water soluble cyanotoxins from freshwater species. Thus an irritant, allergic contact dermatitis may also develop [192]. In addition to the direct or indirect (airborne dermatitis) local effects of cyanotoxins on skin and/or mucous membranes, systemic manifestations were also reported, such as hay fever, asthma and generalized urticarial rash as well as ocular symptoms, e.g., itchy oedematous eyelids with conjunctivitis were frequently seen [103,191,193,194]. Various studies show that Cyanoprokaryota as well as eukaryotic algae may possess allergenic qualities and may act as type I inhalant allergens [195,196,197,198]. Recently abundant airborne filamentous Cyanoprokaryota from India, *Leptolyngbya fragilis (Phormidium fragile)* and *Desmonostoc muscorum* proved to have allergenic potential [23], the latter also known from building surfaces [3].

Moreover, an allergic contact type dermatitis due to cutaneous sensitivity against the accessory photosynthesis pigment phycocyanin of Cyanoprokaryota (*Anabaena* sp.) was registered and confirmed by positive skin patch testing [193]. This could be a very important finding because phycocyanin is commonly a part of the photosynthetic apparatus of Cyanoprokaryota and in Rhodophyceae. Further reports on this kind of sensitization seem lacking, but recently it has been shown that allergies to Cyanoprokaryota may also be caused by non-toxin-containing parts of these organisms even suggesting that Cyanoprokaryota might be an unrecognised ubiquitous allergen [199]. Contrary to that, there are indications of anti-inflammatory, anti-oxidative, cell protective and anti-cancer properties of phycocyanin [200,201,202].

Comprehensively, it can be concluded that there are some algal taxa involved in the triggering of allergies in humans. Whereas aerophytic green algae may be responsible for inhalant allergies, other groups are mainly involved with skin reactions. Although until now there are few medical cases reported, quite some taxa that are recognised as allergenic species are present in the built environment (Table 1). To further elucidate the medical relevance of allergenic algae connected to the built environment, additional studies would be welcome.

## 4. Pathogenic Algae and the Built Environment

It may seem paradoxical to talk about pathogenic algae as they usually are autotrophic, but in special groups of algae, so-called “colourless” forms have evolved. These are living heterotrophic forms and some of them may even act as opportunistic pathogens. Formerly not recognised as related to green algae, the genera *Prototheca* and *Helicosporidium* represent colourless algae that developed to parasitic/pathogenic organisms. Whereas *Helicosporidium* is only known from arthropods so far [203,204], different species of *Prototheca* may lead to infections in mammals and humans [205].

The taxonomic position of *Prototheca*, first described by Krüger in 1894 [206], has been disputed controversially for some time [207,208]. To make it even more complicated, one taxon, formerly assigned to the genus *Prototheca*, *P. filamenta* [209], later known as *Fissuricella filamenta* [210], was revealed to belong to the fungi, now bearing the name *Trichosporon asteroides*, Tremellales, Basidiomycota, according to molecular investigations [211,212,213]. Currently, *Prototheca* is classified among the green algae (Trebouxiophyceae, Chlorophyta) based on ultrastructural and genetic evidence. Its important traits are the occurrence of plastid-like granules in the plasma and the asexual method of reproduction. It is now generally accepted that the genus developed from a *Chlorella*-like form at some point in evolution [214,215,216,217]. In fact, 18S rRNA sequence studies suggest a close affinity with *Auxenochlorella protothecoides,* Trebouxiophyceae [218,219]. According to AlgaeBase, currently 20 species of *Prototheca* are accepted taxonomically [220].

The achloric *Prototheca* species are heterotrophic and therefore require external sources of organic carbon and nitrogen [221]. Their life cycle is similar to that of *Chlorella*-like green algae [222,223], with asexual reproduction with autospores [205,224]. Most of the accepted species are known to only be saprophytic, but some have been found in infections of humans and mammals. *Prototheca cutis*, *Prototheca miyajii*, *Prototheca wickerhamii* and *Prototheca zopfii* have been reported to cause infections in humans [205,208,225], with *Prototheca cutis* [226] and *Prototheca miyajii* [227] only recently described. *Prototheca blaschkeae* is known to be potentially pathogenic only to cows so far [208,228]. *Prothotheca zopfii* may be further divided into subgroups according to ecophysiological traits and molecular characters [225,229,230]. Still many forms and their taxonomic ranks are discussed controversially, a comprehensive monographic treatment still lacking [231].

*Prototheca* spp. are globally ubiquitous and are isolated from various sources, e.g., slime flux of trees, grass, fresh and salt water, wastewater, animals (e.g., cattle, dog, deer), stables (animal buildings), excrements, soil, cow’s milk and other food items (e.g., potato peels, butter, bananas) [205]. Bovine mastitis caused by *Prototheca zopfii* and *Prototheca blaschkeae* represent serious veterinary problems and may result in heavy economic losses to particular dairy farms [228,232]. In humans, protothecosis is rare, and in some cases the aetiology is not clear. The pathogenesis of the so called “protothecosis” is largely unknown, and it is believed that the *Prototheca* species may infect humans through contact with potential sources or by traumatic inoculation [205]. Only in a few cases other than deep traumatic inoculation could it be revealed as way of infection. Recently, it was demonstrated that potentially pathogenic *Prototheca* spp. may form biofilms, and this was brought into context with its ability to cause infections [233]. Viable cells of *Prototheca zopfii*, the species predominant in human infections, were isolated from different samples gained from environmental sources in dairy herds, even from bedding, which can be regarded as an aerophytic and building related source [234]. *Prototheca zopfii* and other pathogens were identified in air samples from semi-closed pig farms evaluated in a study in 2012 [235]. Although in another study, in a serological survey of wastewater workers, professionals who are working in an environment potentially loaded by *Prothotheca* spp. and other pathogens, analysis for *Prototheca* antibody titres (*P. wickerhamii*) was essentially negative [236]. Results of [235] indicate that *Prototheca* spp. may also be spread airborne. The occurrence of protothecosis may be local, with skin as the organ most frequently involved, or disseminated and acute or chronic, with the latter being more common [205]. In many documented infections, patients with compromised immune systems or who are undergoing an immunosuppressive therapy are befallen. Treatment usually involves medical and surgical approaches, but treatment failure is not uncommon. Antifungals such as ketoconazole, itraconazole, fluconazole and amphothericin B are the most common drugs used to date, with amphothericin B as the most promising compound [205].

Furthermore, there have been reported infections of mammals and humans with algae bearing functional (green) chloroplasts, but often the taxonomic nature (species) of the infecting agents could not clearly be retrieved (e.g., [237]: Chlorococcales; [238,239]: Chlorococcales; [240]: *Chlorella* sp.; [241]: *Chlorella* sp.; [242]: *Scenedesmus* sp.; [243]: *Chlorella* sp.; [244]: *Desmodesmus* sp.). Although retrospectively difficult in some cases presented where “Chlorella sp.” was involved, from pictures obtained, it may be possible that *Auxenochlorella protothecoides* or a species of *Myconastes* may have been the etiologic agent. In cases where the causal species was defined very different algal species were involved, such as *Desmodesmus armatus* var. *subalternans* and *Chloroidium saccharophila* [245,246]. The latter species is also abundant in the built environment. In most incidents of “algal infection” (“chlorellosis”) deep traumatic inoculation is almost always involved. Regarding the treatment of green algal infection (“chlorellosis”), the procedure is similar to the treatment of protothecosis with surgical measures and/or drug administration [243,244,246]. Algal infections (*Chlorella* sp., *Chlorochytrium* sp., *Scenedesmus* sp., *Cladophora* sp.) have also been reported in fresh water fish [247] and mussels [248]. In contrary there exists a mutual relationship of the embryos of a salamander with green algae (Chlamydomonadales; until now no official description of the form exists) involving even an intracellular inclusion of the algal partner [249], and there are described various symbioses of protists with green algae [250,251].

House dust and certain wet, warm and detergence influenced surfaces in buildings may be favourable for growth not only for algae but also be a habitat of *Acanthamoeba* spp., free-living amoebae that may act as opportunistic pathogens and lead to eye or even fatal brain infection [252]. Whereas this amoebae often are linked with infections following entry to the eye, some cornea infections have been traced to inefficient and/or contaminated contact lens solutions [253,254,255,256,257]. *Acanthamoeba* may also serve as a vehicle for further pathogenic organismsuch as*Legionella* [252].

Altogether “algal” infections (protothecosis, chlorellosis) are very special incidences, but it becomes clear that algal forms pathogenic to humans also exist in the built environment. However, human infection due to chlorophyllic algae is very rare, but generally, occurrences seem to be more frequent (personal comment, Bradley Ford, M.D. Ph.D., Clinical Associate Professor of Pathology, University of Iowa Hospitals and Clinics).

## 5. Algae Toxic to the Built Environment or to Work of Men

Although the expression “algae toxic to the built environment” may sound a bit harsh, it fits well to the concept of “building pathology” [258]. Usually, characteristics of building material regarding algal colonization are identified in terms of “susceptibility” (how prone a material is to be invaded by algae) or “resistance” (ability to withstand algal “attack”); the expression “bioreceptivity” has also been created [259].

In a 2002 USA survey, direct corrosion-related damages were estimated to cost USD 276 billion a year for the whole US economy, which is 3.1 percent of the USA gross domestic product (GOP) [260]. According to [261], 50% of these damages are due to microbial-induced corrosion (MIC). As algae are a typical component of biogenic crusts forming on the surfaces of buildings and other human structures, their involvement in biodeterioration of these surfaces and materials is under discussion.

The anthropogenic and built environment is very diverse and possesses a lot of different habitats, such as surfaces of buildings, indoor rooms, basements and tunnels, etc., but also recreational areas, parks and water bodies, etc. Analogous to rock inhabiting algae, phototrophs thriving in anthropogenic habitats may colonize niches that correspond to the epilitic, chasmoendolithic, cryptoendolithic and euendolithic lifeforms established on and in natural hard substrates [262]. Manmade underground structures or structures otherwise only irradiated by artificial light may give rise to Lampenflora. Especially external thermal insulation compound systems (ETICS) may be prone to surface colonization as their uppermost layer is thermally decoupled from the wall itself and may lose its warmth during the night and thus fall below the dew point, thereby accumulating liquid water on its surface [263]. Investigations of the initial (primary) growth on the surface of ETICS revealed an algal diversity of more than 70 forms involved [3]. In the initial growth, especially species from the Trebouxiophyceae genera *Chlorella/Chloroidium* and *Diplosphaera/Stichococcus* are abundantly supplemented with a lot of different taxa [3]. On urban surfaces, especially on occasions of runoff water that are fertilized by bird droppings or other nutrient sources, species of *Klebsormidium* may become dominating, especially from the *K. flaccidum* species group [3]. Different studies revealed that surface characters such as surface water absorption, porosity, roughness, hydrophoby/hydrophily of surface, pH and chemical composition of substrate are involved in the growth of building relevant algae and other organisms [3,264,265,266,267,268]. Additionally, external sources of nutrients such as the faeces of animals (e.g., insects, birds) or air pollution may enhance growth [3,269]. Nevertheless, material characteristics are acting together with environmental factors such as temperature, precipitation, exposition and irradiation (light quality and intensity) on the very place of growth (nanoclimate) and surrounding biota (e.g., “infection pressure”—abundance of propagation units, background germ load; allelopathic effects; etc.) and potential external nutrient sources, etc. [3,163]. In Investigations on the initial growth (primary growth) on modern building façades it was evident, that algae are not only utilizing the mere surface of the substrata, but also surface near cracks and pore room [3]. If hidden in cracks and pores, algae capable of growing in reduced light may enjoy a sheltered place with longer lasting moisture.

As there are so many factors that may be involved it is often complicated to reveal which factors promote growth in a single incident. Therefore, further studies on aerophytic algae and other organisms occurring in the built environment are necessary. A valuable tool for ecophysilogic investigations are defined cultures. The collection of building relevant microorganisms, BRMO, established in the course of work on primary colonization of building surfaces, is providing a source of knowledge and documentation for building relevant algae and fungi [1,2,3,31].

Usually it is presumed that algae growing on building surfaces may lower the surface temperature with their transpiration. This may be true for green forms, but many building algae are producing dark colours, which absorb sunlight and convert it to warmth and thereby they may even enhance the surface temperature and increase the thermal stress on the material [270].

Algae growing in anthropogenic habitats may impair the optical conditions of material surfaces, alter the properties of materials as well as chemically change the surface composition, etc., and thus be part of biodeterioration processes. Biological activity on/in building material may lead to chemical alteration of the substrate (e.g., acidification by excretion of acids or hardening by building up of oxalate) and may lead to increased destruction, weathering and increased porosity and permeability of water [159,271]. Oxalate formation, which may result from the concomitant action of microorganisms and environmental conditions, underlines the ambivalent action of many microorganisms on building surfaces, as it may have positive and negative impact. First, as it is a very hard substance it may consolidate the surface but later it may contribute to scaling, especially if ions are dislocated and the material becomes softer just beneath the oxalate layer. If established on delicate paintings it will be always a nuisance, because it is very likely that the structure and colour of the artwork is spoiled [272].

In addition, excreted polymeric substances are of importance. They may be used by the alga as a passive means of collecting and concentrating nutrients and water, but through their repeated swelling and contracting, they may mechanically stress the substrate. Furthermore, calcium carbonate, gypsum or oxalate may be precipitated at gelatinous sheaths [159,272]. Another example is represented by *Klebsormidium*, which is fastened on the surfaces by little gelatinous cushions that are secreted from special points along the trichomes. With its often intense growth, *Klebsormidium* and further accompanying microorganisms may result in profound disfigurement and soiling of the building surface. On the other hand, some species of *Klebsormidium* also possess an interesting biotechnological potential [273].

At surfaces in electrically lit passages in accessible caves there may establish a so-called Lampenflora (eukaryotic algae and blue greens as well as some bryophytes and ferns). This phenomenon is well known [5], but in prehistoric caves with precious wall paintings it is a severe problem. Green algae began to grow on top of the paintings and this cover destroyed the ancient art works: “maladie verte” [274]. As algae usually depend on light for growth the problem seemed easy to fix, and the caves were shut down for some months, the lighting turned off. Afterwards, the surprise was great as the algae seemed to still flourish, and the covered areas had even extended. Some unicellular algae may also grow heterotrophically and use simple organic compounds for their sustenance and thusthis was not such a surprise at all.

There are implications that the colonization of calcareous substrates, such as building materials, by endolithic biocrusts exerts a protective rather than destructive effect against weathering, corrosion and abrasion on carbonate hard substrate surfaces [275], but still, results are controversial whether algae have a direct effect on surface disintegration of buildings and art works or not.

At the moment, it is not yet clear how to best manage surface colonization by algae in the built environment. As we can see, many potential harmful algae (Table 1) exist in the human environment and therefore a prevention or reduction of their occurrence might be desired. On the other hand, in the sense of mitigation of global climate change, we must seek to establish growth of photosynthetic organisms as much as possible. There are already approaches in development that are focussing on the stimulation of favourable plants at buildings and the urban environment and simultaneously avoiding potentially harmful organisms [276].

Some products for building coatings are equipped with certain chemical compounds (biocides) which defend the material against microbial colonization. To reach the target organisms, these compounds must possess a certain solubility, which means that the effect ceases after some time [277,278]. The biocidal approach, still part of the state of the art, is not the most sustainable, and it is not guaranteed that non-target organisms are affected [3,279]. There is evidence that biocides washed out from building structures may be harmful to natural environments or even accumulate in river sediments [280,281]. Various other methods for preventing or removing unwanted algal growth on buildings and cultural heritage have been developed such as the application of photokatalysis, dry ice cleaning, etc. [279,282,283,284]. The most effective way to avoid growth of algae and other microorganisms still is reducting the surface dampness and wetness as much as possible.

Different methods have been developed to assess the stability/susceptibility of building coatings against algal growth [3,265,266,268,285]. In product development, accelerated methods are on demand, but it is challenging to model the huge variety of factors involved [164,286,287,288]. Recently a promising approach was developed that uses microclimate conditions of the most favourable season of the year of a given climate in permanent repetition, thus providing a very practical methodology [263].

The growth of algae on the surface of buildings or work of art is a common phenomenon. Studying the results of various significant work on algae growing on building surfaces reveals that there is occurring a broad variety of potentially harmful algae (Table 1). If these present an actual or even growing medical problem to people, or not, still needs to be investigated. Apart from damaging artwork by obscuring sophisticated artists work, there is still some controversy about the influence of algae on the substrate. If algae growing on building structures are mainly acting as destructive agents or if they act more as protective organisms is debated. As global climate change will alter also local climate conditions this will also have effect on algal growth on building surfaces.

## 6. Potentially Harmful Airborne Algae and Their Abundance in the Built Environment

As a main mode of contact of the human body with harmful algae in the built environment is via inhalation information on airborne algae shall give a basic impression. In order to give an overview on the abundance of potentially harmful algae in the built environment (indoor, Lampenflora, building surfaces), I enlisted airborne algae, potentially toxic and/or allergenic, from literature and set them into context with the built environment (Table 1). This extract is based on a choice of literature, mainly on comprehensive studies or work otherwise important in the theme [3,5,8,10,17,19,23,26,74,107,116,129,130,140,141,142,143,144,145,146,147,148,149,150,151,152,154,155,156,157,158,159,160,161,162,163,164,165,166,167,168,169,170,171,270]. Airborne algae were sampled with different methods, e.g., fan dust sampling, filtration, impaction, impingement, Rotorod sampler, sedimentation, application of a vacuum cleaner and wind nets.

As in the literature, often data from previous work have listed some taxa names as “sensu lato (s.l.)”. Recently, the understanding of many genera has changed (see e.g., [289,290,291,292,293,294,295,296,297,298,299,300,301,302,303,304,305,306]), therefore I applied a broad reception for the mentioned genera. In some of the genera s.l., I specified taxons (new genera names) that were previously included. This does not mean that necessarily all subtaxons of such a genus s.l. are potentially toxic or otherwise harmful. Nevertheless, a recorded occurrence gives the indication that potentially harmful species cannot be ruled out. If there were given species names in the work on airborne algae that were potentially harmful, I took them into the list, giving their previous classification in brackets. Algae names were checked with [30,144,145,146,307]. Additionally, information is given on potential health effects including major toxins produced from toxigenic species [17,26,74,107,116,129,130,143,144,145,146,147,148,149,150,151,152]. Seventy-nine taxa of potentially harmful airborne algae are listed. If all species from the built environment (indoor, Lampenflora, building surfaces) belonging to the mentioned genera or genera s.l., respectively, were listed a much higher figure would apply, but for some of these species there is no information available on their potential health implications so far. With Table 1 it becomes clear that already many aerophytic algae, potentially harmful, have been recorded from the built environment. Some potentially harmful algae have not been recorded from the built environment yet, and this may be because they are bound to their limnic or marine environment and are aerosolised by chance only or they simply have been overlooked so far.

## 7. Conclusions

Although algae occurring in the built environment are not commonly considered to be of great clinical significance, they may be responsible for human disorders. There is growing evidence that algae occurring on buildings or present in the built environment may cause severe health problems in men. Although not common, even toxic events may happen. The allergenic potential of algae emerging in the built environment shall not be neglected as there is ample evidence for allergens in eukaryotic algae and cyanoprokaryota. Pathogenic algae are widespread in limnic and edaphic habitats and have also been recorded for the built environment, though scarce. Organisms that may occur together with aerophytic algae may even have a higher toxic or pathogenic relevance. Finally, there is discussion about the potential of algae potentially toxic to buildings. Therefore, the scientific record still is ambivalent, there is evidence both for detrimental and protecting action from algae to buildings and work of art. Defined strains with documented source from culture collections such as BRMO will be a helpful tool in the future to elucidate the role of harmful algae in the built environment. Further detailed investigations shall reveal the interconnections between the different organisms and habitats. This knowledge will enable the creation of a healthier and more stable environment in habitats dominated and created by men in the future.

## Data Availability

Data sharing is not applicable to this article.
